# Novel Cetacean Morbillivirus in Guiana Dolphin, Brazil

**DOI:** 10.3201/eid2003.131557

**Published:** 2014-03

**Authors:** Kátia R. Groch, Adriana C. Colosio, Milton C. C. Marcondes, Daniele Zucca, Josué Díaz-Delgado, Claudia Niemeyer, Juliana Marigo, Paulo E. Brandão, Antonio Fernández, José Luiz Catão-Dias

**Affiliations:** University of São Paulo, São Paulo, Brazil (K.R. Groch, C. Niemeyer, J. Marigo, P.E. Brandão, J.L. Catão-Dias);; Instituto Baleia Jubarte, Caravelas, Brazil (K.R. Groch, A.C. Colosio, M.C.C. Marcondes);; University of Las Palmas de Gran Canaria, Las Palmas de Gran Canaria, Canary Islands, Spain (D. Zucca, J. Díaz-Delgado, A. Fernández).

**Keywords:** cetacean morbillivirus, Morbillivirus genus, Paramyxoviridae, Guiana dolphin, Brazil, marine mammals, cetaceans, viruses, *Sotalia guianensis*, epizootic outbreaks, South Atlantic Ocean

**To the Editor:** Since 1987, morbillivirus (family *Paramyxoviridae*, genus *Morbillivirus*) outbreaks among pinnipeds and cetaceans in the Northern Hemisphere have caused high rates of death (*1*,*2*). Two morbillivirus species are known to affect aquatic animals: *Phocine distemper virus* (PDV) and *Cetacean morbillivirus* (CeMV). PDV has been isolated from pinnipeds, and 3 strains of CeMV (porpoise morbillivirus [PMV], dolphin morbillivirus [DMV], and pilot whale morbillivirus [PWMV]) have been isolated from dolphins and whales (*3*,*4*). 

Serologic surveys indicate that morbilliviruses infect marine mammals worldwide (*5*); however, only 1 fatal case in a bottlenose dolphin (*Tursiops truncatus*) has been confirmed in the Southern Hemisphere (in the southwestern Pacific Ocean) (*6*). Positive DMV-specific antibody titers in 3 Fraser’s dolphins (*Lagenodelphis hosei*) stranded off Brazil and Argentina in 1999 indicate the exposure of South Atlantic cetaceans to morbillivirus (*7*). We report a case of lethal morbillivirus infection in a Guiana dolphin (*Sotalia guianensis*), a coastal marine and estuarine species that occurs off the Atlantic Coast of South and Central America. 

A female Guiana dolphin calf (108 cm in total body length) (*8*) was found stranded dead in Guriri (18°44’S; 39°44’W), São Mateus, Espírito Santo State, Brazil, on November 30, 2010; the dead calf was severely emaciated. Postmortem examination of the animal showed multifocal ulcers in the oral mucosa and genital slit, diffusely dark red and edematous lungs, and congested and edematous brain. Samples of selected tissues were collected, fixed in buffered formalin, and processed according to routine histopathologic methods. By microscopy, the most noteworthy lesions included marked lymphoplasmacytic and neutrophilic meningoencephalitis, optic nerve perineuritis, and hypophysitis. Lungs showed moderate acute diffuse lymphoplasmacytic and neutrophilic interstitial pneumonia; severe multicentric lymphoid depletion and multifocal necrotizing hepatitis were also observed. 

Immunohistochemistry was performed by using CDV-NP MAb (VMRD, Inc., Pullman, WA, USA), a monoclonal antibody against the nucleoprotein antigen of canine distemper virus that cross-reacts with cetacean morbilliviruses (*9*). Known positive and negative control tissues and test sections with omitted first-layer antibody were included. Viral antigen was detected in neurons in the brain, bronchiolar epithelium and macrophages in the lungs, bile duct epithelium in the liver, and macrophages and lymphocytes in lymph nodes. 

We extracted RNA from frozen lung samples by using TRIzol Reagent (Life Technologies Corporation, Carlsbad, CA, USA) according to the manufacturer’s instructions and amplified a 374-bp conserved fragment of the phosphoprotein (P) gene by reverse transcription PCR. The following *Morbillivirus* spp.–specific primers were used for PCR: 5′-ATGTTTATGATCACAGCGGT-3′ (forward) and 5′-ATTGGGTTGCACCACTTGTC-3′ (reverse) (*3*). MEGA5 (http://megasoftware.net/) was used to construct a neighbor-joining phylogenic tree based on the sequenced amplicon from this study (GenBank accession no. KF711855) and 12 other GenBank sequences that represent the 6 morbillivirus species already described in the literature. The analysis placed the Guiana dolphin strain at the CeMV clade, but segregated it from the already described dolphin morbillivirus strains PMV, DMV, and PWMV (Figure). The sample shared 79.8% nt and 58.4% aa identity with PMV, 78.7% nt and 56.6% aa identity with DMV, and 78.7% nt and 57.1% aa identity with PWMV. Within the *Morbillivirus* spp., PDV shared the lowest sequence identity (51.1% nt and 26.8% aa). 

**Figure:**
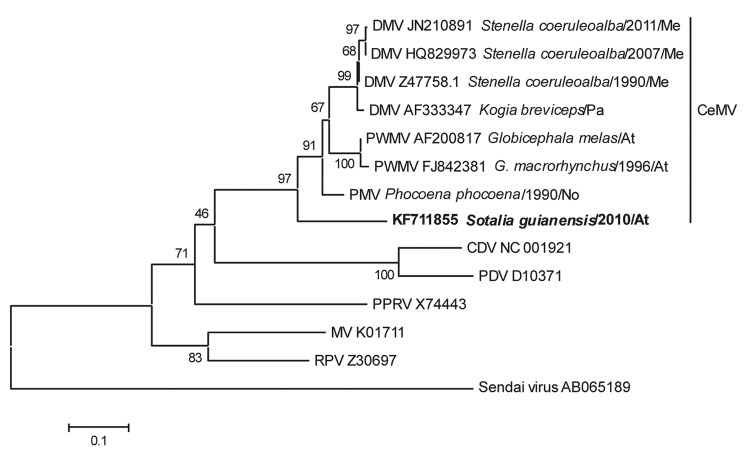
Phylogenetic tree of a 374-bp conserved region from the phosphoprotein gene of a cetacean morbillivirus isolated from a Guiana dolphin (in boldface; GenBank accession no. KF711855) and 12 other previously described morbilliviruses. Sendai virus was added as an outgroup member. Sequences were aligned and a neighbor-joining tree with 1,000 bootstrap replications was generated by using MEGA5 (http://megasoftware.net/). For comparison, recognized viruses of the *Morbillivirus* spp. (*Measles virus* [MV], *Rinderpest virus* [RPV], *Peste-des-petits ruminants virus* [PPRV], *Canine distemper virus* [CDV], and *Phocine distemper virus* [PDV]) were included, as were the 3 *Cetacean morbillivirus* (CeMV) strains: porpoise morbillivirus (PMV), dolphin morbillivirus (DMV), and pilot whale morbillivirus (PWMV). Sequence names are followed by species of cetacean, year of stranding (when available), and the abbreviation for the geographic area. Me, Mediterranean Coast; Pa, Pacific Ocean; At, Atlantic Ocean; No, North Sea. The sequence for PMV strain *Phocoena phocoena*, is from Barrett et al. (*3*). The scale bar indicates nucleotide substitutions per site.

In summary, sequence analysis of the morbillivirus from the dead Guiana dolphin suggests that the virus is a novel strain of the CeMV species; this conclusion is supported by phylogenic analysis and geographic distribution of the virus and by its distinct host. Emaciation, marked lymphoid depletion, interstitial pneumonia, and meningoencephalitis are common findings in morbillivirus-infected animals (*1*,*2*). Together with antigenic and genomic evidence, our findings indicate that morbillivirus infection is extant in Guiana dolphins in the waters off Brazil. 

Morbillivirus outbreaks have caused a high number of deaths among pinnipeds and cetaceans and are a major risk to previously unexposed nonimmune populations of aquatic mammals (*1*,*2*). A high number of morbillivirus-related deaths have not yet been reported among aquatic mammals in the waters off Brazil, but our findings shows that Guiana dolphin calves are susceptible to infection. Subclinical morbillivirus infection with immune suppression has been reported in bottlenose dolphins in Florida (*10*). It is unknown whether subclinical infection occurs in this host population or whether the virus has undergone species-adaptive changes, as proposed for PWMV (*4*). The sequence data from our study suggest that the virus from the Guiana dolphin calf is the fourth member of the CeMV group and is closer to the root of the CeMV clade than to that of DMV, PMV, or PWMV. Further studies are required to determine the epidemiology of morbillivirus infection in this and other cetacean species and to assess the risk for epizootic outbreaks among South Atlantic cetaceans.
